# Further delineation of defects in MRPS2 causing human OXPHOS deficiency and early developmental abnormalities in zebrafish

**DOI:** 10.1038/s41431-025-01858-1

**Published:** 2025-05-13

**Authors:** Amoolya Kandettu, Mayuri Yeole, Hamsini Sekar, Kishore Garapati, Namanpreet Kaur, Aakanksha Anand, Pranavi Hegde, Karthik Nair, Raghavender Medishetti, Vivekananda Bhat, Periyasamy Radhakrishnan, Suneel C. Mundkur, Hebbar A. Shrikiran, Akhilesh Pandey, Aarti Sevilimedu, Sanjiban Chakrabarty, Anju Shukla

**Affiliations:** 1 Department of Public Health Genomics, Manipal School of Life Sciences, Manipal Academy of Higher Education, Manipal, India; 2 Department of Medical Genetics, Kasturba Medical College, Manipal, Manipal Academy of Higher Education, Manipal, India; 3 Center for Innovation in Molecular and Pharmaceutical Sciences, Dr. Reddy’s Institute of Life Sciences, University of Hyderabad Campus, Gachibowli, Hyderabad India; 4 Department of Laboratory Medicine and Pathology, Mayo Clinic, Rochester, MN USA; 5 Manipal Academy of Higher Education, Manipal, India; 6 Institute of Bioinformatics, International Technology Park, Bangalore, India; 7 Department of Paediatrics, Kasturba Medical College, Manipal, Manipal Academy of Higher Education, Manipal, India; 8 Center for Individualized Medicine, Mayo Clinic, Rochester, MN USA; 9 Center for Rare Disease Models, Dr. Reddy’s Institute of Life Sciences, University of Hyderabad Campus, Gachibowli, Hyderabad India

**Keywords:** Medical genetics, Proteomics

## Abstract

Mitochondrial ribosomal protein-small 2 (*MRPS2*) encodes a vital structural protein essential for assembling mitoribosomal small subunit and thus mitochondrial translation. Any defect in mitochondrial translation impacts OXPHOS activity and cellular respiration. Defects in *MRPS2* have been implicated recently in four families with combined oxidative phosphorylation deficiency-36 (MIM# 617950). We herein describe two individuals from two unrelated families with variable phenotypes of acute onset severe metabolic decompensation and symptomatic hypoglycemia. Exome sequencing identified bi-allelic variants in *MRPS2* (NM_016034.5) in the affected individuals: P1: c.490 G > A p.(Glu164Lys); and P2: c.413 G > A p.(Arg138His). Further evaluation of the variant c.490 G > A p.(Glu164Lys) in patient-derived skin fibroblasts revealed decreased expression of *MRPS2* transcript and protein levels of MRPS2 along with expression of complex I and IV proteins. Proteomics analysis revealed decreased expression of small subunit proteins and increased expression of large subunit proteins. Also, reduced complex I and IV enzyme activities, mitochondrial respiration (OCR), and altered mitochondrial morphology on confocal imaging were observed. Additionally, *mrps2* knockout zebrafish larvae demonstrated an abnormal developmental phenotype and reduced Complex IV activity. With these findings, we identify additional families with variants in *MRPS2*, illustrating the variable clinical spectrum and validate the pathogenicity of defects in MRPS2 through in-vitro and in-vivo assays.

## Introduction

Mitochondrial translation is executed by a dedicated translation machinery that includes many regulatory factors and mitochondrial–specific ribosome subunits or the mitoribosomes. The mitoribosome consists of two subunits: a 28S small subunit (mtSSU) composed of 30 mitoribosomal proteins (MRPs) and a 12S rRNA, while the 39S large subunit (mtLSU) is composed of 52 MRPs, 16S rRNA, mt-tRNA^Val^ and mt-tRNA^Phe^ [1, 2]. The mtLSU functions as a catalyst for peptide bond formation and attaching the ribosome to the inner mitochondrial membrane, whereas mtSSU aids in decoding the mRNA and further recruits a correct aminoacyl tRNA to the translating ribosome [3]. Mitochondrial translation is responsible for synthesizing 13 proteins of the OXPHOS system, encoded by mtDNA. Hence, translational defects in mitoribosomes have been implicated in combined OXPHOS complex deficiency [4].

MRPs, the structural components of the mitoribosome, are encoded by the nuclear genome and imported into mitochondria to facilitate mitoribosome assembly [5]. To date, variants in 14 MRP encoding genes including four MRPL genes - *MRPL3* (MIM*607118), *MRPL12* (MIM*602375), *MRPL24* (MIM*611836), *MRPL44* (MIM*611849) and ten MRPS genes i.e. *MRPS2* (MIM*611971), *MRPS7* (MIM*611974), *MRPS14* (MIM*611978), *MRPS16* (MIM*609204), *MRPS22* (MIM*605810), *MRPS23* (MIM*611985), *MRPS25* (MIM*611987), *MRPS28* (MIM*611990), *MRPS34* (MIM*311994) and *PTCD3* (MIM*614918) have been implicated in human mitochondrial disorders. Though pathogenic variants in these genes have been associated with variable phenotypes [6], most affected individuals present with severe, early-onset multi-organ dysfunction and are often associated with a reduced lifespan [7].

The mitochondrial ribosomal protein S2 encoded by *MRPS*2 (MIM*611971) is an evolutionarily conserved protein and an integral component of the mtSSU. Biallelic variants in *MRPS2* have been recently reported to cause combined oxidative phosphorylation deficiency 36 (COXPD36, MIM# 617950), presenting with variable phenotypes of global developmental delay, ataxia, hypotonia, severe lactic acidosis, hypoglycemia, and sensorineural hearing loss in four unrelated individuals till date [8–10].

We herein describe the phenotypic and genotypic details of two unrelated individuals with biallelic variants in *MRPS2*. We show the impact of the novel variant in one of the individuals on the mitochondrial RNA and protein expression, OXPHOS activity, morphology, and function. We also demonstrate that loss of *mrps2* leads to delayed development and abnormal developmental phenotypes in zebrafish.

## Methodology

### Participant recruitment

We recruited two probands (P1 and P2) from two unrelated families of Indian origin as part of an ongoing study on rare genetic disorders. Informed consents approved by the institutional ethics committee were obtained from the recruited families.

### Genetic testing

The genomic DNA was extracted from whole blood using DNeasy Blood and Tissue Kits (QIAGEN, Valencia, California). Singleton exome sequencing (ES) was performed using TWIST Bioscience capture kit for P1 and P2 on an Illumina NextSeq Platform (Illumina, Inc. San Diego CA) and analysis was performed as described previously [11]. Validation and segregation analysis was performed by Sanger sequencing for prioritized variants in both families.

### In silico protein modeling and analysis

In silico protein analysis was performed for the identified missense variants in both the families. Small mitoribosome subunit structure (PDB ID: 7PO3) was obtained from protein data bank (RCSB PDB) (https://www.rcsb.org/structure/7PO3; accessed on 27 January 2024). Visualization of protein structure was performed on Chimera 1.16 [12]. In silico mutagenesis for MRPS2 protein was performed and alterations in polar contacts in mutant protein were analyzed as compared to wild type. Additionally, any alterations in polar contacts of MRPS2 protein with other interacting small mitoribosome subunits were also analyzed. Multiple sequence alignment (MSA) was performed using Clustal Omega [13].

### Cell culture

Dermal fibroblasts were obtained from skin biopsy of P1 and the normal controls (C1 and C2). Fibroblast cells were cultured in Dulbecco’s modified Eagle’s medium (DMEM) (Gibco, USA) supplemented with 10% fetal bovine serum (FBS) (Gibco, USA). Fibroblasts were enzymatically passaged in 0.25% Trypsin-EDTA (HiMedia, India). All cultures were maintained in 37 °C incubators at 5% CO_2_. The culture medium was replenished every two days until cells became 85% confluent. The cells were used up to 4 passages only. Further, total RNA was isolated from skin fibroblasts using the TRIzol method. The RNA quality was assessed by A260/280 ratios and converted to cDNA using SuperScript™ IV VILO™ Master Mix.

### Proteomics analysis

P1-derived and control fibroblasts (volunteer donor; GM05659 and GM08333, Coriell Institute) were cultured in complete medium with Minimum Essential Medium. Cell lysate-derived tryptic peptides were labeled with tandem mass tags (TMT; Thermo Fisher Scientific, A44520) as per the manufacturer’s protocol. The P1-derived samples were used in triplicate. Following a label check, pooled peptides were fractionated by basic pH reversed-phase liquid chromatography (bRPLC) on a C18 column, and twenty-four fractions were analyzed by liquid chromatography-tandem mass spectrometry (LC-MS/MS) for total proteomics with modifications [14].

### Intracellular ATP estimation

For ATP quantification, 1×10^6^ fibroblast cells were seeded and lysed with passive lysis buffer. Intracellular ATP levels were determined in 5uL of cell lysate using the ATP determination kit (Invitrogen, USA) as per the manufacturer’s guidelines. The data were normalized against the total protein concentration [15].

### Zebrafish F0 knockout generation

The F0 knock out of *mrps2* in zebrafish was generated using CRISPR/Cas9 mediated gene editing, as described previously [16].

To generate the *mrps2* crispants, chemically modified synthetic guide RNAs of the most efficient sgRNAs (sg1 and sg2, sourced from Synthego) (Supplementary Table S2) were injected at 1.2 ng/embryo sgRNA mix with 800 pg Cas9 protein, per embryo.

### Statistical analysis

Statistical analysis was performed using GraphPad prism. ANOVA and Student unpaired t-test and data were represented as mean ± SD, and a *p*-value less than 0.05 was considered statistically significant. Cell-based assays were repeated thrice and performed in duplicates. The zebrafish experiments were performed in three to five sets of injectants, each with *n *> 70 per group.

*Detailed methodology for immunoblotting, mitochondrial complex activity, oxygen consumption rate, mitochondrial morphology analysis, and mitochondrial membrane potential is available in the supplementary information.

## Results

### Clinical findings

**Fig. 1:**
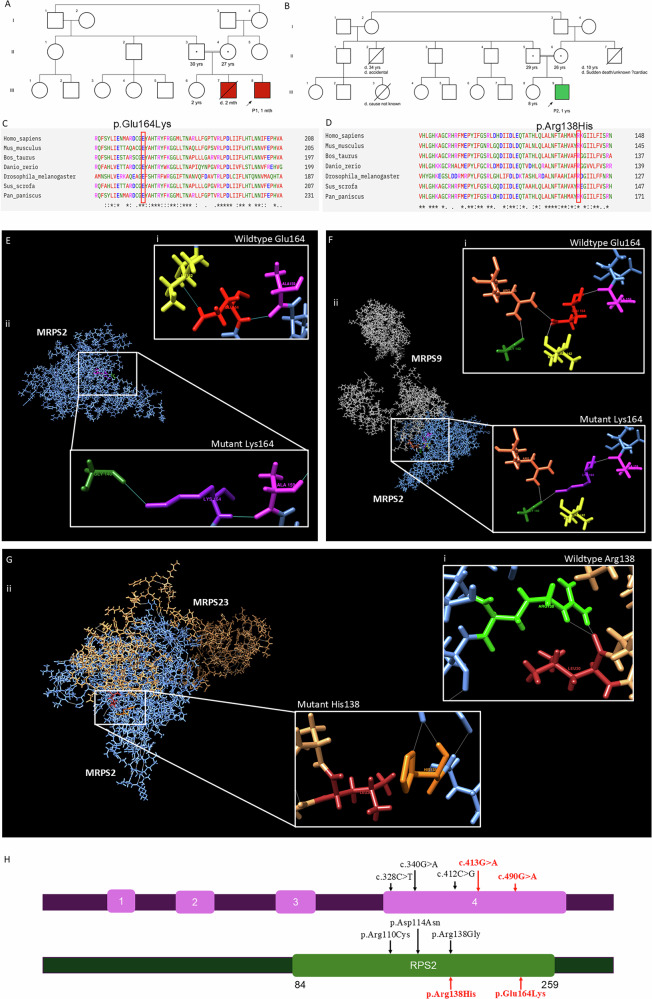
Details of family history and variants in *MRPS2*. Pedigree of (**A**) Family 1 and (**B**) Family 2. Multiple sequence alignment shows (**C**) amino acid glutamine at 164 position and (**D**) arginine at 138 position is evolutionary conserved among Homo sapiens, Mus musculus, Bos taurus, Danio rerio, Drosophila melanogaster, Sus scrofa and Pan paniscus. **E** In silico protein modelling in MRPS2 protein structure shows (**i**) polar contacts (blue lines) of wild type Glu164 residue (red) with Ile142 (yellow) and Ala159 (pink) and in silico mutagenesis revealed that (**ii**) mutant Lys164 residue (purple) lost polar contact with Ile142 instead formed polar contact with Gly140 (dark green). **F** In silico protein modelling of MRPS2 and MRPS9 protein structures shows (**i**) wild type Glu164 residue (red) forms polar contacts (white lines) with Arg145 residue (coral) of MRPS9 and in silico mutagenesis revealed (**ii**) absence of polar contacts between mutant Lys164 residue (purple) of MRPS2 and any other residue of MRPS9. **G** In silico protein modelling of MRPS2 and MRPS23 protein structures shows (**i**) wild type Arg138 residue (light green) forms polar contacts (white lines) with Leu30 residue (brown) of MRPS23 and in silico mutagenesis revealed (**ii**) absence of polar contacts between mutant His138 (orange) of MRPS2 and any other residue of MRPS23. **H** Schematic representation of MRPS2 (NM_016034.5) and protein showing our variants (highlighted in red) and previously reported variants. RPS2:Ribosomal protein S2.

P1 was born out of a consanguineous union (Fig. 1A) with unremarkable antenatal and perinatal history. He presented with refusal to feed, excessive crying, hurried breathing, poor peripheral circulation, and weak respiratory efforts on day 41 of life. There was a family history of a deceased elder sibling with similar complaints at two months of age. On clinical examination, his weight was 3.2 kg (−2.51 SD), length 48 cm (−3.68 SD), and head circumference 36.5 cm (−1.22 SD). The arterial blood gas analysis on admission was suggestive of severe metabolic acidosis, serum lactate level was >140 mg/dl (<27 mg/dl), blood creatinine phosphokinase was 631 U/L (46–171 U/L), and liver enzymes (SGOT – 129 IU/L; SGPT – 84.9 IU/L) were mildly elevated. 2D echo evaluation was suggestive of severe pulmonary arterial hypertension, dilated right atrium, and right ventricle with moderate tricuspid regurgitation. Tandem mass spectrophotometry (TMS) from blood showed a mild derangement of the acyl-carnitine profile, whereas the gas chromatography-mass spectrophotometry (GCMS) of urine was suggestive of significantly elevated ethyl malonic acid. He recovered from this episode of decompensation and was discharged after ten days of hospital admission. He was re-evaluated at six months and later at 13 months of age and was noted to have normal development and no recent episode of decompensation.

P2 is a one-year and five-month-old male born of a consanguineous union of family 2 (Fig. 1B). He was born of a normal vaginal delivery, and the perinatal period was uneventful. He developed generalized tonic-clonic type seizure at the age of one month. He was hospitalized at an external center where the random blood glucose was documented to be 12 mg/dl (70–150 mg/dl). The other hematological and biochemical investigations were within normal limits for his age. During admission, he had three more episodes of convulsions following a similar semiology and was further started on antiepileptics. The GCMS and TMS was performed, however, the report was not available for him. He was discharged in a stable condition and did not develop any further events of hypoglycemia-induced seizures. On evaluation at our center at one-year and five-months of age, he had been seizure-free for the past one year and was developmentally normal. His length was 78 cm (−1.46 SD), weight was 7.9 kg (−2.86 SD), and head circumference was 47 cm (−0.23 SD). Biochemical investigation revealed a random blood sugar level of <41 mg/dl whereas the other investigations were within normal limits. He had mild fullness of cheeks, mild tenting of vermilion of the upper lip, and multiple Mongolian spots over the lower lumbar region. On per abdomen examination, the liver was palpable 3 cm below the right costal margin and soft in consistency. The rest of the systemic examination was unremarkable.

### Exome sequencing identifies bi-allelic variants in *MRPS2*

Singleton exome sequencing of P1 revealed a novel missense variant c.490 G > A p.(Glu164Lys) in exon 4 of *MRPS2* (NM_016034.5) (NC_000009.12:g.135503732 G > A) in a homozygous state. This variant is observed in 26 individuals in heterozygous state in the gnomAD database (v4.1.0). Exome analysis for P2 identified a previously reported missense variant c.413 G > A p.(Arg138His) in exon 4 of *MRPS2* (NM_016034.5) (NC_000009.12:g.135503655 G > A).

This variant is observed in 48 individuals in heterozygous state in the gnomAD database (v4.1.0). These variants are not observed in our in-house dataset of 3200 exomes. In silico tools such as Mutationtaster, CADD Phred and ClinPred were consistent in predicting the variants to be damaging to the protein function. Bi-allelic segregation by Sanger sequencing confirmed the carrier status of the parents of P1 and P2. The variant c.490 G > A p.(Glu164Lys) was classified as variant of uncertain significance and the variant c.413 G > A p.(Arg138His) was classified as pathogenic variant as per the American College of Medical Genetics and Genomics guidelines [17]. MSA performed using Clustal Omega showed the variant c.490 G > A and c.413 G > A being highly conserved across species (Fig. 1C, D).

### In silico protein modeling and analysis

In silico mutagenesis of the p.Glu164Lys variant in MRPS2 protein structure revealed that the wildtype Glu164 forms polar contacts with Ile142 and Ala159 whereas mutant Lys164 forms polar contacts with Gly140 and Ala159 (Fig. 1E). Additionally, alterations in the interacting residues of MRPS2 with other small subunits of mitoribosomes revealed that mutant Lys164 resulted in the loss of polar interaction between MRPS2 and MRPS9 structures (Fig. 1F) On the other hand, in silico mutagenesis of the second variant p. Arg138His revealed no alterations of interactions with other residues of MRPS2. On analysis, it was observed that the wildtype residue Arg138 forms polar contacts with Leu30 residue of MRPS23 protein, however, mutant His138 does not form any polar contacts with any residue of MRPS23 (Fig. 1G).

### Functional analysis of the variant, c.490G>A p.(Glu164Lys) in *MRPS2*

**Fig. 2:**
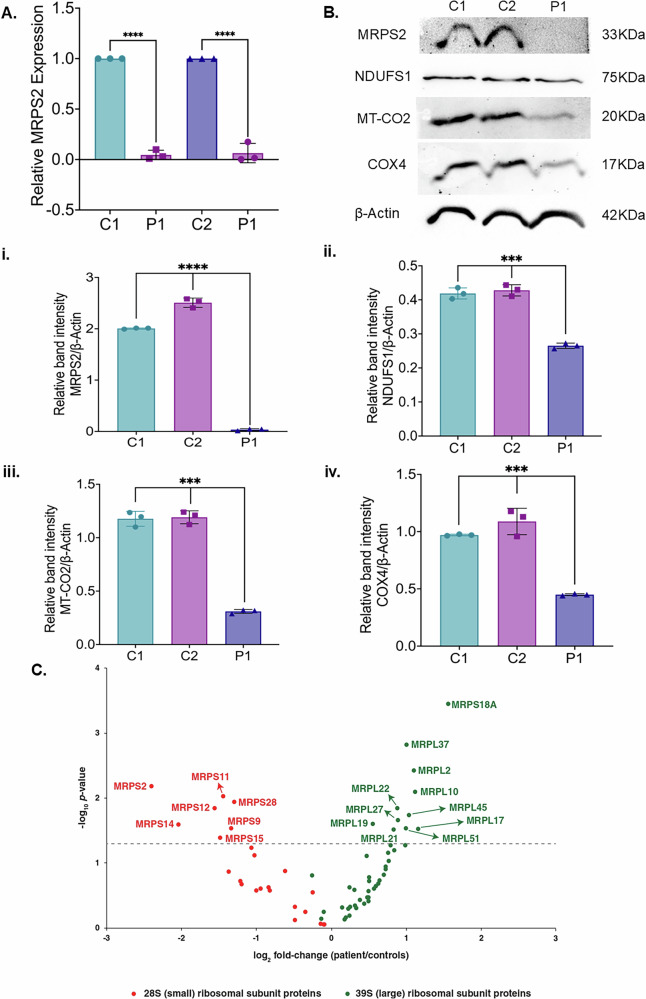
Analysis of MRPS2 expression and OXPHOS proteins in controls and P1 patient derived cells. **A** qRT-PCR analysis showing relative *MRPS2* mRNA expression in control 1 (C1), control 2 (C2) and patient (P1) cell lines. Expression of *MRPS2* in P1 was significantly upregulated when compared to C1 and C2. β-Actin was used as the internal control; **B** Representative blot images for protein expression of MRPS2, NDUFS1, MT-CO2 and COX4; (**i-iv**) Western blot analysis of protein expression of MRPS2, NDUFS1, MT-CO2 and COX4 in C1, C2 and P1 fibroblast cell lines. Densitometric analysis was performed upon normalization of MRPS2, NDUFS1, MT-CO2 and COX4 protein band intensity to respective β-Actin band. *p < 0.05, **p < 0.01, ***p < 0.001 and ****p < 0.0001; **C** Altered abundance of mitochondrial ribosomal proteins. A volcano plot showing alterations in the levels of various mitochondrial ribosomal subunit proteins. Small and large mitochondrial ribosomal subunit proteins are represented by red and green dots, respectively, as indicated. The horizontal dashed line corresponds to *p *= 0.05.

**Fig. 3:**
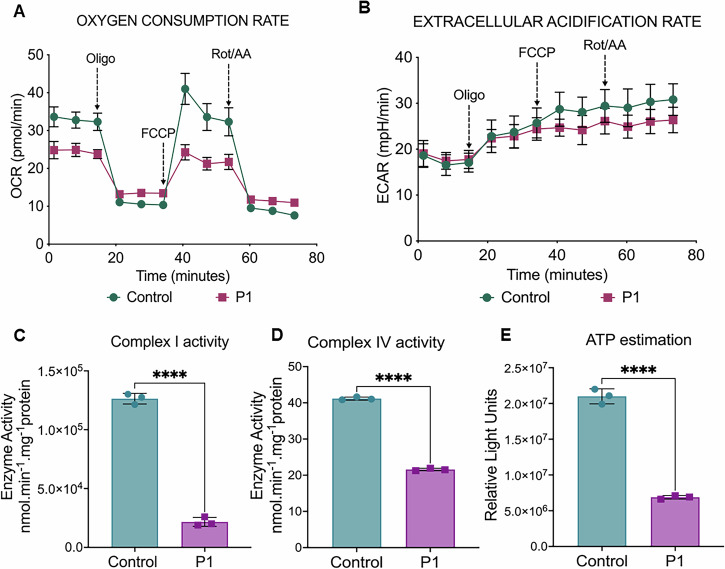
Analysis of mitochondrial OCR and EACR in control and patient fibroblast cells. **A** Representative graph showing the OCR in control and P1 fibroblast cell lines. Injection of oligomycin, FCCP, and antimycin A and rotenone (A/R) are indicated (**B**) Representative graph showing the EACR in control and P1 cell lines. Injection of oligomycin, FCCP, and antimycin A and rotenone (A/R) are indicated (**C**) Mitochondrial complex I enzymatic activities in MRPS2 control and P1 cell lines (**D**) Mitochondrial complex IV enzymatic activities in control and P1 cell lines (**E**) Representative graph showing intracellular ATP levels. ATP determination kit was used to quantify intracellular ATP levels. *p < 0.05, **p < 0.001, ***p < 0.0001 and ****p < 0.00001.

The molecular effect of the variant c.490 G > A was investigated by using primary skin fibroblast from P1 and appropriate control samples (C1 and C2). Quantitative analysis of *MRPS2* expression by qRT-PCR showed reduced expression in P1 (Fig. 2A) compared with both the control samples (C1 and C2). Quantitative estimation of proteins by western blot revealed a significant reduction in MRPS2 as well as complex I (NDUFS1) and complex IV (MT-CO2, COX4) subunits in P1 (Fig. 2B (i-iv)). On proteomics analysis, a significant decrease in MRPS2, NDUFS1, and COX4 expression was noted in patient P1 when compared to controls (Supplementary Fig. 1A–C). Although the levels of MT-CO2 were also found to be decreased by proteomics analysis, this was not statistically significant (fold-change: 0.48, *p *= 0.2) (Supplementary Fig. 1D). All the detected small subunit components were found to be decreased, and most large subunit proteins were increased in P1 compared to controls (Fig. 2C). Analysis of cellular respiration by Seahorse XF24 Extracellular Flux Analyzer showed decreased OCR and increased extracellular acidification rate (ECAR), suggesting perturbed mitochondrial respiration capacity in fibroblast cells from P1 (Fig. 3A, B, Supplementary Fig. 2). We also observed reduced OXPHOS enzyme activity in P1, suggesting perturbed mitochondrial OXPHOS defect (Fig. 3C, D). A significant decrease in ATP levels in fibroblast cells of P1 was also observed when compared to control fibroblast cells suggesting perturbed mitochondrial ATP synthesis (Fig. 3E).

Control and P1 mitochondria were visualized using MitoTracker Red staining and analyzed using confocal microscopy. Analysis of mitochondrial morphology and network showed reduced branch length in P1 mitochondria compared to control fibroblast mitochondria (Supplementary Fig. 3A, B). Staining for MMP with Rhodamine123 showed decreased fluorescence intensity in P1 compared to the control fibroblast cells, indicating reduced mitochondrial membrane potential (Supplementary Fig. 3C, D).

### Loss of *mrps2* in zebrafish impacts early development and mitoribosome subunits

**Fig. 4:**
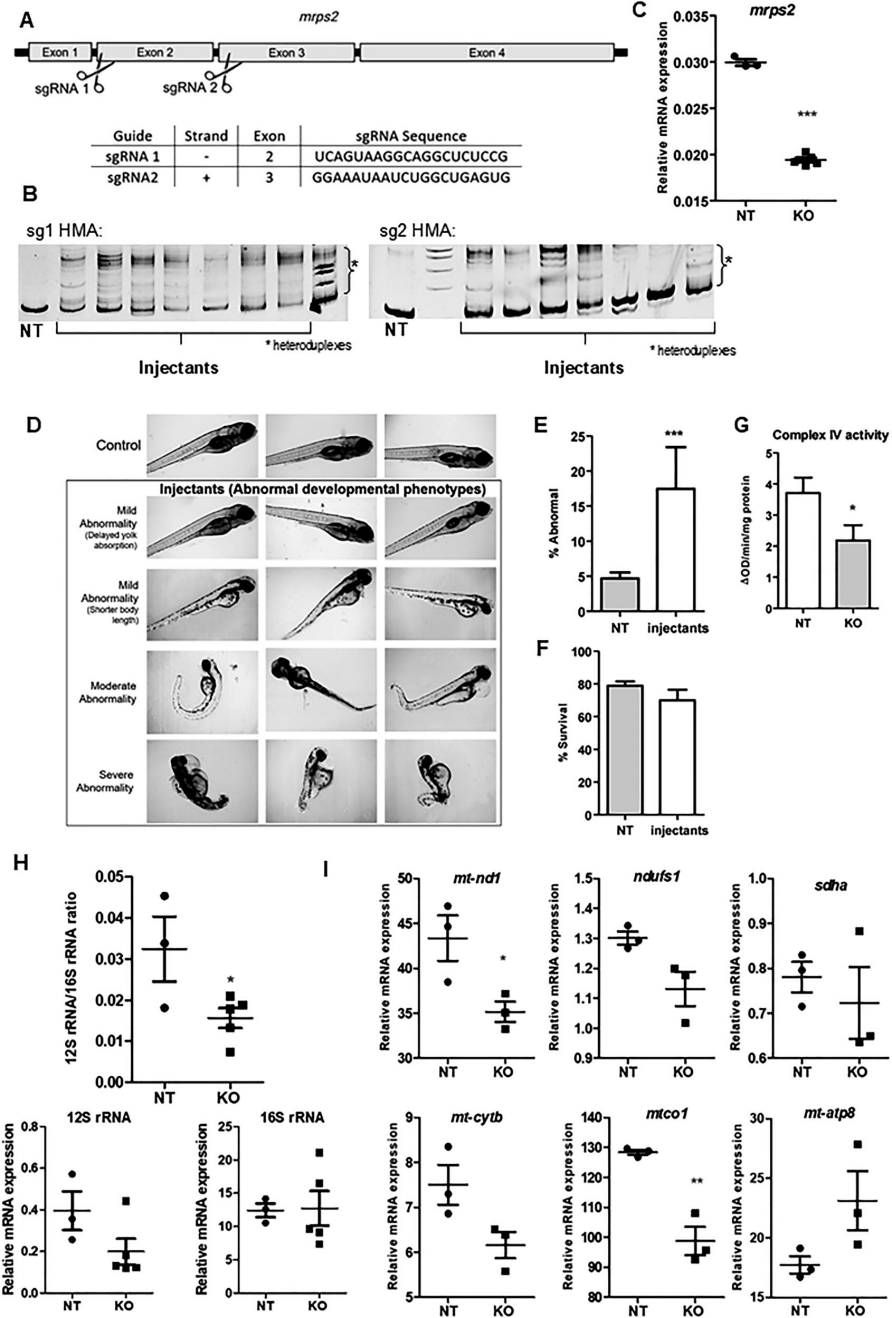
Phenotypes in the *mrps2* knockout zebrafish larvae. **A** A schematic of the zebrafish *mrps2* with the location of the selected sgRNA target sites indicated. The sequences of the two sgRNAs used in the study are provided in the table, **B** Assessment of editing efficiency for each sgRNA at 24 h post injection, by a heteroduplex mobility assay (HMA), **C** Relative expression levels of the *mrps2* mRNA in the controls and injectants (crispants), **D** Representative bright field images of wild-type and *mrps2* crispants (at 3dpf) illustrating abnormal developmental phenotypes observed in the crispants, as labeled, **E**, **F** Quantification of the number of larvae showing abnormal development at 5dpf (**E**) and survival at 7dpf (**F**) (5 experiments, NT *n *= 560, KO *n *= 820), **G** Complex IV activity measurement in mitochondria isolated from control and *mrps2* crispants, **H** 12S to 16S rRNA ratio in the crispants compared to controls. Results from 3 to 5 independent experiments are quantified, and error bars represent SEM, (**I**) Relative mRNA levels of OXPHOS subunits genes including *nd1*, *ndufs1* (Complex I), *sdha* (Complex II), *cytb* (Complex III), *mt-co1* and *mt-co2* (Complex IV), in the *mrps2* crispants compared to controls.

To uncover the impact of a loss of *mrps2* function during early development, we used the F0 knockout strategy. Guides targeting exons 2 and 3 were chosen based on editing efficiency and injected together to create F0 crispants (Fig. 4A). Extensive editing was observed at each guide target site, as shown in the representative image of the HMA PCR assay (Fig. 4B). Due to the lack of availability of an antibody that recognizes zebrafish Mrps2, we measured and observed a significant decrease in the mRNA levels of *mrps2* in the crispants, presumably due to nonsense-mediated decay of the edited mRNA (Fig. 4C). The crispants and control larvae were raised to 7dpf with observation of developmental abnormalities daily. A subset of the *mrps2* crispants showed abnormalities such as delayed or absent hatching, delayed yolk absorption, bent body or tail, severe oedema and dysmorphisms between 3 and 5dpf (Fig. 4D, E). As the number of injectants with biallelic genomic edits leading to a complete loss of function is likely to be low, the fraction of larvae that showed these abnormalities was also low. However, over five independent experiments, the number of larvae with abnormal phenotypes was consistently and significantly greater in the *mrps2* crispants as compared to the NT (control) injectants. No significant differences in survival were observed until the age of 7dpf (Fig. 4F). In order to assess mitochondrial function, Complex IV activity was measured in mitochondria isolated from control and *mrps2* crispants, and a slight reduction in activity was observed (Fig. 4G), similar to that detected in the patient fibroblasts. We also observed a decrease in the 12S rRNA levels in the *mrps2* crispants, resulting in a significant reduction in the 12S/16S rRNA ratio (Fig. 4H), as observed in fibroblasts of P1, indicating a destabilization of the mitoribosome [8]. Further, transcript levels of mitoribosome complex subunits (complex I, IV, and V) were found to be reduced in the crispants (Fig. 4I).

## Discussion

Combined oxidative phosphorylation deficiency is an autosomal recessive multisystem disorder with variable manifestations resulting from a defect in the mitochondrial oxidative phosphorylation (OXPHOS) system. Previously, four variants in *MRPS2* have been reported in four unrelated individuals (Fig. 1H) [8–10]. The current report expands the phenotypic and genetic spectrum of *MRPS2-*related COXPD36 (MIM# 617950). The clinical presentations of P1 and P2, though variable, overlap with those of COXPD36 as well as several other OXPHOS protein defects. P1, who presented with early onset of metabolic decompensation and multisystem dysfunction, did not suffer any further episodes till the end of infancy. However, there is a history of early demise of his sibling with a similar episode in early infancy. Such acute episodes leading to early mortality have not been reported in other individuals with COXPD36. The clinical presentation of P2 with symptomatic hypoglycemia closely resembled the other reported individuals with COXPD36. Unfortunately, hearing evaluation could not be carried out for both P1 and P2 and is planned in their follow-up visit as sensorineural hearing loss has been documented in all four reported individuals [8–10] to date. Neuroimaging findings were unremarkable in individuals reported by Gardeitchik et al. [8] and Papadopoulos et al. (2023), however neuroimaging records were not available for individuals reported by Liu et al. [9] as well as current probands. Both P1 and P2 at present appear to have non-progressive symptoms as has been observed in other individuals with this condition. Clinical, biochemical, and genomic variant details of the present and reported individuals with *MRPS2* defects have been provided in Table 1.

**Table 1:** Comparison of previously reported individuals with our study.

	This study		Gardeitchik et al. [8]		Liu et al. [9]	Papadopoulos et al. [10]
Family	Family 1	Family 2	Family 3	Family 4	Family 5	Family 6
Proband ID	P1	P2	P3	P4	P5	P6
**Gender**	Male	Male	Female	Male	Female	Female
**Ethnicity**	Indian	Indian	Austrian	Tunisian	Chinese	Sri Lankan
**Consanguinity**	Consanguineous	Consanguineous	Non-consanguineous	Non-consanguineous	Not known	Consanguineous
**Variants in** ***MRPS2*** **(NM_016034.5)**	c.490 G > A p.(Glu164Lys)	c.413 G > A p.(Arg138His)	c.[328 C > T];[340 G > A] p.[(Arg110Cys)]; [(Asp114Asn)]	c.413 G > A p.(Arg138His)	c.412 C > G p.(Arg138Gly)	c.328 C > T p.(Arg110Cys)
**Exon/Intron**	Exon 4	Exon 4	Exon 4	Exon 4	Exon 4	Exon 4
**Zygosity**	Homozygous	Homozygous	Compound heterozygous	Homozygous	Homozygous	Homozygous
**Age at last examination**	6 months	1 year 5 months	11 years	11 years	7 years	5 years
**Age at onset**	Day 41	1 month	1 year	6 years	4 years 5 months	17 months
**Complaints at initial evaluation**	Cardiogenic shock	Hypoglycaemia	Failure to thrive	Hypoglycaemia	Hypoglycaemia	Hypoglycaemia
**Developmental history**						
**Motor delay**	Mild	No	Yes	Yes	No	Yes
**Cognitive delay**	No	No	Yes	Yes	Yes	Yes
**Behavioural difficulties**	No	No	No	No	Yes	Yes
**Speech delay/disorder**	No	No	Yes, due to sensorineural hearing loss	Yes, due to sensorineural hearing loss	Yes, due to sensorineural hearing loss	Yes, due to sensorineural hearing loss
**Clinical examination**						
**Failure to thrive**	Yes	No	Yes	Yes	Yes	Yes
**Dysmorphism**	No	No	Yes	No	No	Yes
**Hypotonia**	No	No	Yes	Yes	No	Yes
**Cardiac involvement**	Yes	No	No	No	No	No
**Investigations**						
**MRI (Brain)**	NA	NA	Normal	Normal	NA	Normal
**Cardiac evaluation**	Severe Pulmonary arterial hypertension on presentation	NA	NA	NA	NA	NA
**Hearing evaluation**	NA	NA	Sensorineural hearing loss	Sensorineural hearing loss	Sensorineural hearing loss	Sensorineural hearing loss
**Biochemical testing**						
**Hypoglycaemia**	No	Yes	Yes	Yes	Yes	Yes
**Plasma lactate (mmol/l)**	Elevated	Normal	Elevated	Elevated	Elevated	Elevated
**Creatinine Kinase**	Elevated	NA	No	No	NA	No
**Increased expression of Krebs intermediate (Urine)**	Significantly elevated ethyl malonic acid	NA	Yes	Yes (2-oxoglutarate in urine)	No	Yes (Fumarate/Malate)
**OXPHOS complex activity**	Fibroblast: CI: low CIV: low	NA	Muscle: CI: normal, CIV: low Liver: CI: low, CIV: low Fibroblasts: CI: normal; CIV: low	Muscle: CI: normal, CIV: low Liver: CI: low, CIV: low Fibroblasts: CI: normal, CIV: low	Not done	Fibroblasts: CI: normal CIV: low

*MRI* magnetic resonance imaging, *MRS* magnetic resonance spectroscopy, *EEG* electroencephalogram, *CSF* cerebrospinal fluid, *NA* not available, *CI* complex I, *CIV* complex IV.

MRPS2 is a crucial structural component of the small subunit (mt-SSU) of the mitoribosome and plays an essential role in decoding and recruitment of mitochondrial messenger RNAs (mt-mRNAs). The intricate role of MRPS2 in mitoribosome assembly and mitochondrial translation underscores its importance in cellular bioenergetics and highlights the potential consequences of its dysfunction. It has been demonstrated that the loss of individual MRPS proteins leads to decreased 12S rRNA steady-state abundance, indicating the interdependence of these components for proper mitoribosomal function. In silico analysis suggested that the variant p.Glu164Lys would affect the intramolecular interactions within the MRPS2 protein as well as intermolecular interactions with other MRPS proteins which might lead to an unstable protein and overall, the mt-SSU. Concurrent to this, in our study, fibroblasts from P1 showed reduced mRNA and protein expression of MRPS2 when compared to control cells. Reduced MRPS2 expression level impacted mitochondrial protein translation with decreased mitochondrial OXPHOS proteins NDUFS1 (Complex I component), MT-CO2, and COX4 (Complex IV components), suggesting combined mitochondrial OXPHOS defect in P1 cell lines. In silico analysis for previously reported variant p.Arg138His, did not reveal any alterations within the MRPS2 protein structure. However, an absence of the interaction between mutant MRPS2 protein and MRPS23 was observed which is likely to impact mitoribosome assembly and thus mitochondrial protein synthesis as observed in patient fibroblasts in the previous study [8]. Additionally, to investigate the impact of the variant observed in P1 on individual subunits of the complexes, we performed proteomics analysis using LC-MS/MS. The findings are in keeping with a previous report where MRPS2-deficient fibroblasts were shown to have normal levels of the large subunit proteins on a background of impaired small subunit assembly [8] and likely indicate overall changes in mitoribosomal component synthesis and assembly in association with decreased availability of MRPS2.

The decreased assembly of mt-SSU and the resultant lack of functional mitoribosomes impairing mitochondrial translation due to defects in MRPS2 are likely to cause multiple OXPHOS deficiencies. Previously, Papadopoulos, T. et al. (2023) reported normal activities for complexes I, II, and III, but a marked decrease in the amount of complex IV and its enzymatic activity in *MRPS2* mutant fibroblasts. We also found reduced enzymatic activities of mitochondrial complex I and IV in *MRPS2* mutant cell lines along with depleted intracellular ATP levels. The functional consequences of the *MRPS2* variant on mitochondrial function were further supported by altered bioenergetics in cell lines of P1. A decreased OCR and an increase in EACR indicated impaired mitochondrial respiration in mutant cells. Conversely, the increased ECAR reflected a shift towards glycolysis, suggesting metabolic adaptations in response to mitochondrial dysfunction caused by the *MRPS2* variant. Perturbation in mitochondrial OXPHOS is known to reduce mitochondrial membrane potential [18]. Concurrently, Rhodamine123 staining showed decreased fluorescence intensity in cell lines of P1 as compared to control cells, indicating reduced membrane potential due to mitochondrial OXPHOS defect. Mitochondrial OXPHOS defect is also linked to altered mitochondrial structure. Imaging analysis showed reduced mitochondrial branch length, suggesting fragmented mitochondria in fibroblasts of P1.

Furthermore, we used the zebrafish model to evaluate the impact of *mrps2* loss on the mitoribosome and early organ development. The crispants (F0 knockouts) are genetically mosaic but contain a stable genetic edit facilitating phenotypic studies from the larval to the adult stages. The F0 strategy is effective in measuring the direct consequences of the loss of a critical protein without the added layer of genetic adaptation or compensation that arises during the generation of a stable knockout line [19–21]. Previously, few human mitoribosomal proteins with orthologues in zebrafish have been investigated for their impact on developmental phenotypes and mitochondrial dysfunction. A morpholino mediated knockdown of Mrpl24 resulted in locomotor impairment and structural defects, arising from the impaired mitoribosome assembly [22]. A role for Mrpl4 in notch signaling was reported using the zebrafish model, however the authors did not comment on any mitochondrial or developmental phenotypes in the knockout [23]. In our studies in the *mrps2* crispants, we found a significant decrease in the 12S rRNA levels, indicating the destabilization of the mitoribosome in these larvae, accompanied by changes in the expression of OXPHOS subunit genes and a reduction in Complex IV activity. This is likely the basis of the developmental delay in the crispants, seen in the form of delayed hatching and yolk absorption, shorter body length, oedema and generalized dysmorphisms. Also, similar to the observations in the case of *mrpl24* morphants, we did not observe any impact on survival or a major locomotion deficit in the crispants (Supplementary Fig. 4), possibly due to the remaining mitoribosome activity and function.

In conclusion, we report additional individuals with variants in *MRPS2*, thus expanding the phenotypic and genotypic spectrum of COXPD36. We also demonstrate the pathogenicity of the novel variant added to this study through extensive in-vitro and omics assays performed on patient fibroblasts. We also demonstrate the impact of loss of *mrps2* in zebrafish leading to severe developmental abnormalities.

## Supplementary information

MRPS2 supplementary material

## Data Availability

The mass spectrometry proteomics data have been deposited to the ProteomeXchange Consortium via the PRIDE partner repository with the dataset identifier PXD062317. The additional data supporting this study’s findings are available from the corresponding authors upon reasonable request.
