# Supplementary material for: Further delineation of defects in MRPS2 causing human OXPHOS deficiency and early developmental abnormalities in zebrafish
Source: Eur J Hum Genet. 2025 May 13;33(11):1451–61. doi: 10.1038/s41431-025-01858-1 (PMC12583485; doi:10.1038/s41431-025-01858-1)
Supplement: Supplementary file 1 — MRPS2 supplementary material [file 41431_2025_1858_MOESM1_ESM.docx]

**Further delineation of defects in MRPS2 causing human OXPHOS deficiency and early developmental abnormalities in zebrafish**

Amoolya Kandettu^1 *^, Mayuri Yeole^2 *^, Hamsini Sekar^3^, Kishore Garapati^4,5,6^, Namanpreet Kaur^2^, Aakanksha Anand^2^, Pranavi Hegde^1^, Karthik Nair^2^, Raghavender Medishetti^3^, Vivekananda Bhat^2^, Periyasamy Radhakrishnan^2^, Suneel C. Mundkur^7^, Shrikiran A. Hebbar^7^, Akhilesh Pandey ^4,5,8^, Aarti Sevilimedu^3,9#^, Sanjiban Chakrabarty^1#^, Anju Shukla^2#^

*^1^Department of Public Health Genomics, Manipal School of Life Sciences, Manipal Academy of Higher Education, Manipal, India*

*^2^Department of Medical Genetics, Kasturba Medical College, Manipal, Manipal Academy of Higher Education, Manipal, India*

*^3^Center for Innovation in Molecular and Pharmaceutical Sciences, Dr. Reddy’s Institute of Life Sciences, University of Hyderabad Campus Gachibowli, Hyderabad, India*

*^4^Department of Laboratory Medicine and Pathology, Mayo Clinic, Rochester, Minnesota, USA*

*^5^Manipal Academy of Higher Education, Manipal, India*

*^6^Institute of Bioinformatics, International Technology Park, Bangalore, India*

*^7^Department of Paediatrics, Kasturba Medical College, Manipal, Manipal Academy of Higher Education, Manipal, India*

*^8^Center for Individualized Medicine, Mayo Clinic, Rochester, Minnesota, USA*

*^9^Center for Rare Disease Models, Dr. Reddy’s Institute of Life Sciences, University of Hyderabad Campus Gachibowli, Hyderabad, India*

*****Equally contributing first authors

^#^ **Corresponding authors:**

Dr Anju Shukla

Email id: anju.shukla@manipal.edu

ORCID: 0000-0001-8938-4941

Dr Sanjiban Chakrabarty

Email id: sanjiban.c@manipal.edu

ORCID: 0000-0002-6018-8098

Dr Aarti Sevilimedu

Email id: AartiS@drils.org

ORCID: 0000-0003-2856-0213

**Supplementary Material:**

**cDNA conversion and qRT-PCR**

Total RNA was isolated from the controls (C1 and C2) and subject (P1) fibroblast cell lines using TRI Reagent (Molecular Research Centre, USA) following the manufacturer's protocol. Subsequently, 2 µg of total RNA was converted to cDNA using High-Capacity cDNA Reverse Transcription kit (Applied Biosystems, USA), as previously described [1]. Gene expression analysis by qRT- PCR was performed using PowerUp SYBR Green Master Mix (Applied Biosystems, USA) for gene expression analysis. ACTB gene was used as an internal control and fold change for the paired samples was calculated. Primer sequences for MRPS2 and ACTB genes are given in supplementary table S2.

**Immunoblotting**

Cells were lysed with RIPA buffer supplemented with protease inhibitor cocktails, quantified using Bradford method (Sigma-Aldrich, USA) and 30-50µg of protein was resolved on 10-12% SDS-PAGE. Further, proteins were transferred onto 0.45µm nitrocellulose membrane (Bio-Rad, USA), blocked with 5% non-fat dry milk (HiMedia, India) for respective proteins, and incubated for 1 hour at room temperature. The blots were then incubated separately with respective primary antibodies for overnight at 4°C [MRPS2 (1:1000) (Invitrogen, USA); NDUFS1 (1:3000) (ABclonal, Wuhan, China); MT-CO2 (1:2500) (Invitrogen, USA); COX-IV (1:3000) (Cell Signaling Technologies, USA); Beta-Actin (1:5000) (Cell Signaling Technologies, USA)]. Horseradish peroxidase (HRP)-conjugated secondary anti-rabbit antibody (1:10000) (Jackson ImmunoResearch Labs, USA) incubation was performed at RT for 2 hours. Further, ECL substrate (BioRad Laboratories, USA) was used to visualize the bands using iBright 1500 (Invitrogen, USA). ImageJ was used for densitometric analysis, and all proteins were normalized to Beta-Actin, the loading control.

**LC-MS/MS analysis**

Briefly, peptides were separated by liquid chromatography on a PepSep column using a flow rate of 350 nl/min over a 150-minute reversed-phase gradient, followed by mass spectrometry analysis by an Orbitrap Ascend mass spectrometer. Data acquisition was carried out in MS3 with real time search. Precursors were isolated at a window of 0.7 m/z and those with charge states ranging from +2 to +6 were considered for MS/MS events. Ions were fragmented with a normalized collision energy at 35% and analyzed in the ion trap. For real time search, spectra were searched against the human proteome database followed by the synchronous selection of 10 fragments for further fragmentation by MS3. Ions were isolated with an isolation window of 2 m/z and fragmented with collision energy of 65%, and fragment ions were analyzed in the Orbitrap. Database search was performed using Sequest in Proteome Discoverer 3.0 with reviewed human protein sequences from UniProt (20,432 entries) as the protein sequence FASTA file. Identification was done at the MS2 level using fully tryptic cleavage specificity, with TMTPro at lysine and peptide N-terminal set as fixed modifications. Quantification was done at MS3 level using relative intensities of the TMT reporter ions. Fold-changes of molecules were calculated as average values from patient replicates over average values from control samples. A t-test was carried out to obtain p-values.

**Analysis of mitochondrial complex activity**

The mitochondrial complex-I activity was measured in mitochondria-enriched cell lysate using an assay mixture containing sodium azide, antimycin A, ubiquinone, and NADH (Sigma-Aldrich, USA; prepared in 100mM phosphate buffer). Complex-I activity was measured spectrophotometrically at 340nm using an Infinite 200 PRO multimode reader (Tecan, Switzerland). The specificity of the complex-I activity was monitored by using 1mM Rotenone (complex-I specific inhibitor). The mitochondrial complex-IV activity was measured in mitochondria-enriched cell lysate using 1mM reduced cytochrome C (Sigma-Aldrich, USA; prepared in 100mM phosphate buffer). Complex-IV activity was checked spectrophotometrically at 550nm using an Infinite 200 PRO multimode reader (Tecan, Switzerland). The specificity of the complex-IV activity was monitored by using 10 mM NaN3 (complex-IV specific inhibitor).

**Oxygen consumption rate (OCR) analysis**

Fibroblast cells were seeded at a density of 1x10^4^ cells per well in complete media. The cells were incubated overnight at 37°C and 5% CO_2_. OCR and ECAR values were determined on the Seahorse XFp (Agilent, USA) by running the cell culture miniplates. The media was replaced 1 hour prior to reading with the test assay medium supplemented with pyruvate, glutamine, and glucose. The injections were in the following order - oligomycin, FCCP, and Rotenone/antimycin A. Each experiment was performed in duplicates.

**Analysis of mitochondrial morphology**

For analysis of mitochondrial morphology and network, 4.5 x 10^4^ control and P1 fibroblast cells were plated on 3 mm μ-Dish (Ibidi, Germany). After 48 hours, cells were washed with cold PBS and incubated with 100nM of MitoTracker Red (Molecular Probes, USA) for 30 mins at 37°C in dark. After incubation, cells were washed with cold PBS twice and imaged using a Leica SP8-DMi8 confocal microscope. ImageJ was used to quantify mitochondrial morphology. Quantitative analysis of mitochondrial network morphology was performed using ImageJ with macro toolset, MiNA (Mitochondrial Network Analysis) [2]. Different ROIs (individual cells) were selected from the original image and cropped out. The image was then preprocessed in the following order: unsharp mask, CLAHE (Enhance Local Contrast), and median filtered. This was followed by producing a simplified morphological model for image analysis by first converting the individual cell images into binary thresholding. The binary images were further processed by applying the following commands: Skeletonize and Analyze Skeleton (2D/3D). The tool then identifies and calculates the branch length and provides the output as a table. For each group, 25 cells were analyzed to determine the mitochondrial morphology in control and P1.

**Analysis of mitochondrial membrane potential (MMP)**

To quantify mitochondrial membrane potential, 4.5 x 10^4^ fibroblast cells were plated on 3mm μ-Dish (Ibidi, Germany). After 48 hours, cells were washed and stained with 100nM of Rhodamine-123 (MMP, Molecular Probes, USA) and incubated for 30 mins at 37°C in dark. Cells will then be washed with PBS twice and live imaged using a Leica SP8-DMi8 confocal microscope. ImageJ was used to quantify mitochondrial membrane potential. From control and P1, 25 individual cells were selected using the freehand ROI tool. Each cell was then analyzed for fluorescence intensity using the measure command. Intensity values were plotted for control and P1 cell lines using GraphPad Prism.

**Single guide RNA (sgRNA) design and testing**

The F0 knock out of *mrps2* in zebrafish was generated using CRISPR/Cas9 mediated gene editing. Target regions were chosen in exon 2, exon 3, and exon 4 of the gene using CHOPCHOP and Synthego CRISPR design tools. A non-target sequence not found in the zebrafish genome was chosen as a control (NT) [3]. The sg RNA template for each target was ordered as single-stranded DNA with the addition of T7 promoter at 5′ end and tail oligo sequence. The sequences of guide targets and sgRNA templates are listed in supplementary table S1. Guide RNAs were synthesized by in vitro transcription as described previously [4,5]. The in vivo editing efficiency of each sgRNA was tested by microinjection of the gRNA-Cas9 complex into one-cell stage embryo followed by a Heteroduplex Mobility Assay (HMA) at 24hpf. For the final experiments, chemically modified synthetic guide RNAs of the most efficient sgRNAs (sourced from Synthego) were injected.

**Heteroduplex Mobility Assay (HMA)**

A Heteroduplex Mobility assay was performed for visualizing gene edits post microinjections in 24hpf embryos. Briefly, embryo lysates were made by heating them in 50mM NaOH followed by neutralization with 1M Tris pH 8.0. 0.1-0.3 µl of the lysate was used as input for the PCR using primers spanning the target site. As mentioned, 8 injectants and 1 control (NT) were genotyped. The PCR products were resolved using native PAGE and DNA bands were visualized after staining.

**Microinjections:**

For microinjections for the F0 study, *mrps2* synthetic guide RNA mix (sg1+sg2) or NT gRNA was allowed to form a complex with Cas 9 protein by incubating at 37°C for 10min. This RNP complex was microinjected (effective concentration of guides 1.2ng per embryo along with 800pg of Cas 9 protein) in one cell stage embryos (>100/group), followed by incubation of the embryos at 28ºC. The indels were visualized using the heteroduplex mobility assay (HMA) on 10% native PAGE after 24 hours post microinjection. Differential amplicon migration as compared to wild-type control indicated the presence of indels and confirmed effective editing *in vivo*.

**Phenotypic characterization of the *mrps2* F0 knockout zebrafish larvae:**

Development was monitored on a daily basis and the number of deaths were recorded. Phenotypes were observed and imaged at 5dpf using bright-field microscopy (EVOS cell imaging system) using 4x magnification and as per the published methodology [6].

**Quantitative real-time qPCR analysis (zebrafish)**

Total RNA was isolated using Trizol from 50 larvae at 3dpf for each individual batch of injectants in n=3 to 5 experimental sets, consisting of three control injectants and five mRPS2 injectants. Larvae were pooled independent of phenotypes, for each group. cDNA was synthesized with 600ng of total RNA using PrimeScript™ RT reagent Kit (RR037A-Takara) in a reaction volume of 10µl. qPCR was performed using TB Green mix on QuantStudio5 (Applied Biosystems). Data was analyzed using Ct method (∆∆Ct) and normalized to *rpl13a* or RNAPD (*polr2d*) reference gene. 0.2ul of cDNA was used per reaction with zebrafish-specific exonic primers for each gene (Table S2). 0.2ul of 1:10 diluted or 1:100 diluted cDNA was used per reaction for 12S (*mt-rnr1),* 16S *(mt-rnr2)* and 18S rRNA genes. Relative gene expression was plotted for nontargeting (NT) injectant control and mRPS2 guide injectants using Graph Pad Prism.

**Complex IV Activity Assay (zebrafish):**

Complex IV activity was measured using the Complex IV Human Enzyme Activity Microplate Assay Kit (Abcam – ab109909) according to the manufacturer’s protocol (without antibody enrichment). Isolated mitochondria (3 micrograms sample per reaction) was used as input for the assay. Mitochondrial were isolated from 30 larvae (7dpf) as described in Sengupta et.al, 2021 [7].

**Locomotion analysis:**

Locomotion analysis was performed as described in [8]. Briefly, the Zebrabox system and Zebralab software were used. Single larvae (7dpf) were gently placed in a 24 well plate (in 1ml of E3) 1hr before the experiment and placed inside the Zebrabox for acclimatization for 10 minutes. For the light dark locomotion assay, locomotion was recorded over 10min dark, followed by three alternative light/dark cycles of 3 minutes each. The integration period was set to 1 min; distance moved was calculated for each minute. Parameters evaluated included total distance, average speed, total movement duration and inactivity time. The data obtained was analysed using Microsoft Excel and graphed using Graphpad prism (n=40/group).

**Table S1: Primer sequence details for gene expression analysis**

| **No.** | **Primer name** | **Primer sequence (5'- 3')** | **No. of bases** | **Product size** | **Purpose** |
| --- | --- | --- | --- | --- | --- |
| **1** | **MRPS2_For** | AGCAGGTTGAGGCTCTCTAT | 20 | 133 | Gene expression  (cell lines) |
|  | **MRPS2_Rev** | CTTGGCTGTGGTTTGCTTTG |  |  |  |
| **2** | **B-Actin_For** | ATGATCTGGGTCATCTTCTC | 20 | 131 |  |
|  | **B-Actin_Rev** | CGGGCAGTCAAGGTTTTACA |  |  |  |
| **3** | **HMA sg1_Fwd** | TTATCGTATCACCCTGGCATTT | 20 | 281 | Genotyping  (zebrafish) |
|  | **HMA sg1_Rev** | CAGACTCACCGGTTTCTATGG | 21 |  |  |
| **4** | **HMA sg2_Fwd** | CCCTCACTCAGCCAGATTATTT | 22 | 209 |  |
|  | **HMA sg2_Rev** | CGTATCAGTCATCTGACACCAA | 22 |  |  |
| **5** | **HMA sg3_Fwd** | TCTGATGGAGCCGTATCTCT | 20 | 319 |  |
|  | **HMA sg3_Rev** | GTGCTCCTGGAAGACGTTATT | 21 |  |  |
| **6** | **HMA sg2.2_Fwd** | CTTACAATGAGAATGTAACTTGTGCAG | 27 | 201 |  |
|  | **HMA sg2.2_Rev** | CATGTCGGCAGCCTTTCTT | 19 |  |  |
| **7** | **rpl13a_Fwd** | TGGTGAGGTGTGAGGGTATCAAC | 23 | 298 | Gene expression (zebrafish) |
|  | **rpl13a_Rev** | AATTTGCGTGTGGGTTTCAGAC | 22 |  |  |
| **8** | **Ube2a_Fwd** | TGACTGTTGACCCACCTTACAG | 22 | 265 |  |
|  | **Ube2a_Rev** | CAAATAAAAGCAAGTAACCCCG | 23 |  |  |
| **9** | **mrps2_Fwd** | CCTGTCATGGACAGACTTTGAG | 22 | 140 |  |
|  | **mrps2_Rev** | GAAGAGCTCAGACAAGTGGAAATA | 24 |  |  |
| **10** | **mt-nd1_Fwd** | GCCTACGCCGTACCAGTATT | 20 | 111 |  |
|  | **mt-nd1_Rev** | TCCTCGGGGGCCTATTACAT | 20 |  |  |
| **11** | **ndufs1_Fwd** | TCCTCTCTTCAATGCACGTATC | 22 | 108 |  |
|  | **ndufs1_Rev** | CACCCAGATGGTTGTAAGAGTAG | 23 |  |  |
| **12** | **Sdha_Fwd** | CCGTCAGTTGCATTTCTCCAT | 20 | 114 |  |
|  | **Sdha_Rev** | GCTCCAACCACGACTGCATCAA | 22 |  |  |
| **13** | **mt-cytb_Fwd** | CGGCTGACTTATTCGGAGCAT | 21 | 172 |  |
|  | **mt-cytb_Rev** | GACGTAGCCCACAAAAGCTGTT | 22 |  |  |
| **14** | **mt-co1_Fwd** | GGAATAGTAGGGACCGCATTAAG | 23 | 110 |  |
|  | **mt-co1_Rev** | AAAGCATGGGCAGTAACAATAAC | 23 |  |  |
| **15** | **mt-atp8_Fwd** | ATGCCTCAGCTTAATCCAAA | 20 | 136 |  |
|  | **mt-atp8_Rev** | TGTGCTCTTTAGCATCAACTT | 20 |  |  |
| **16** | **mt-rnr1_Fwd (12s)** | AGCAGAGTGTCTCTTTGAACC | 20 | 105 |  |
|  | **mt-rnr1_Rev(12s)** | CCCTTGTCTGTGCTTCTGTATTA | 23 |  |  |
| **17** | **mt-rnr2_Fwd(16s)** | CAAAGACGATCCGGCCTATAA | 22 | 115 |  |
|  | **mt-rnr2_Rev(16s)** | TCCAACATCGAGGTCGTAAAC | 21 |  |  |
| **18.** | **18s rrna_Fwd** | ATCACCAAGCAGGAGTACGA | 20 | 80 |  |
|  | **18s rrna_Rev** | TACGGCTGAGAGACTGAGAGA | 21 |  |  |

**Table S2**

| sgRNA Target | sgRNA oligo |
| --- | --- |
| g1 (Exon 2) | TCAGTAAGGCAGGCTCTCCG |
| g2 (Exon 3) | CTTGTGTCCAAGATGGACGC |
| g2.1 (Exon 3) | GGAAATAATCTGGCTGAGTG |
| g3 (Exon 4) | TCCAGTACAATCCTGGCGTC |
| NT | GGAGGCGTTCGGCCACAG |


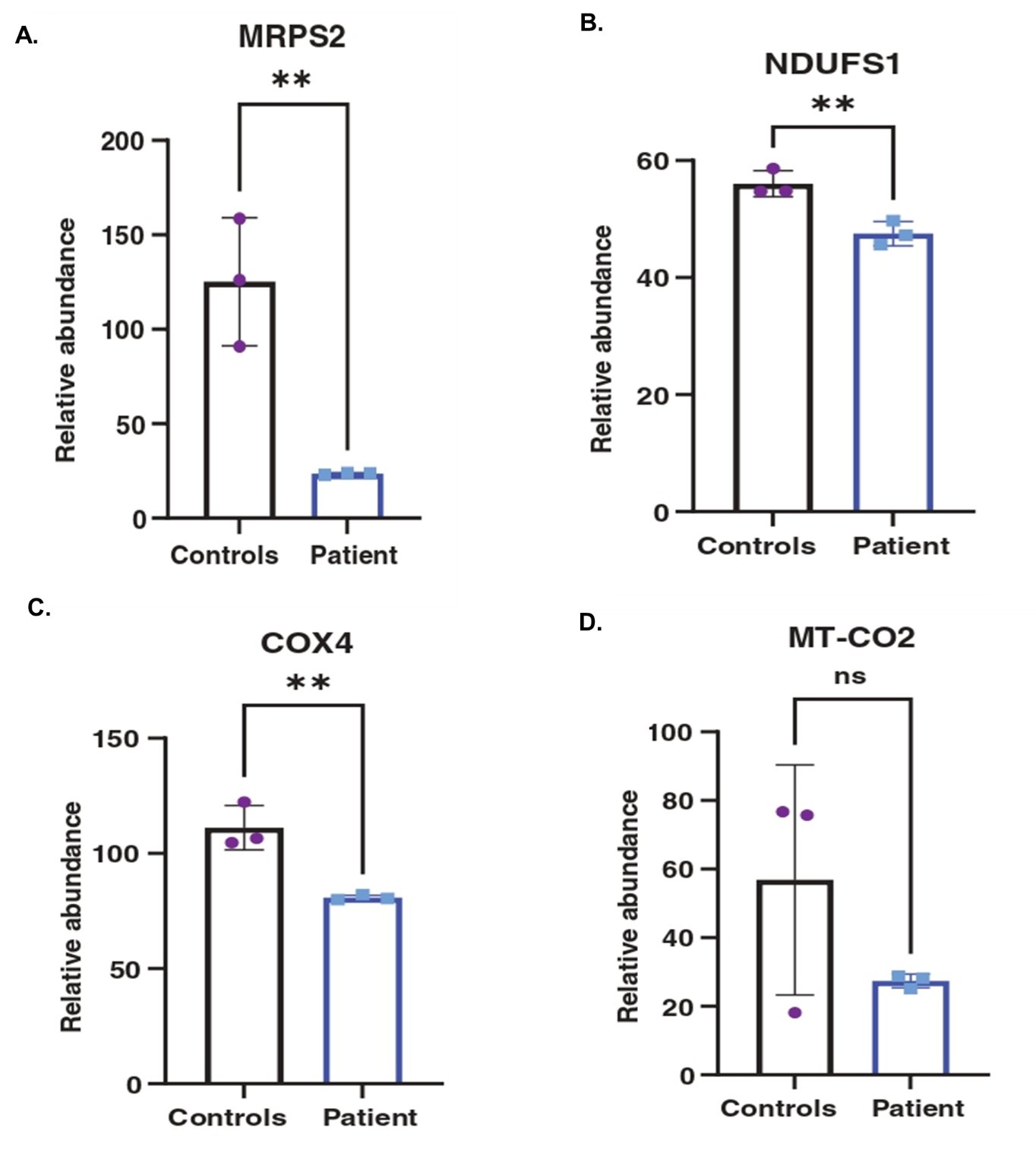


**Supplementary Figure 1: Proteomics analysis of MRPS2-related patient fibroblasts.** Bar graphs showing the relative abundance of mitochondrial proteins from tandem mass tag (TMT)-based relative quantitation across P1 and control samples. **(A)** MRPS2 **(B**) NDUFS1 **(C)** COX4 and **(D)** MT-CO2. **: p<0.01; ns: p>0.05


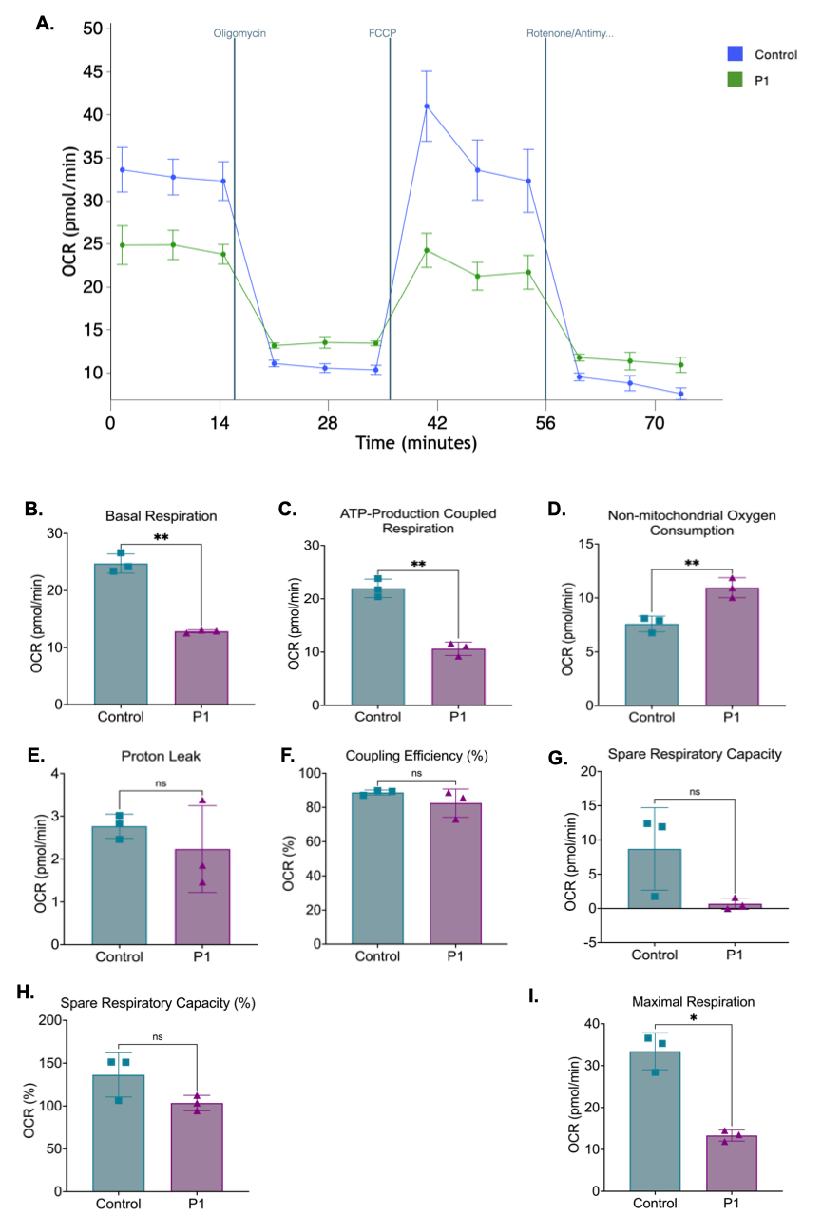


**Supplementary Figure 2: (A)** Representative seahorse instrument generated graph showing the OCR in control and P1 cell lines. **(B-I)** Representative bar graph showing quantification of different mitochondrial bioenergetic parameters: basal respiration, ATP-linked respiration, non-mitochondrial respiration, proton leak, coupling efficiency, spare respiratory capacity and maximal respiration. *p<0.05, **p<0.001, ***p<0.0001.


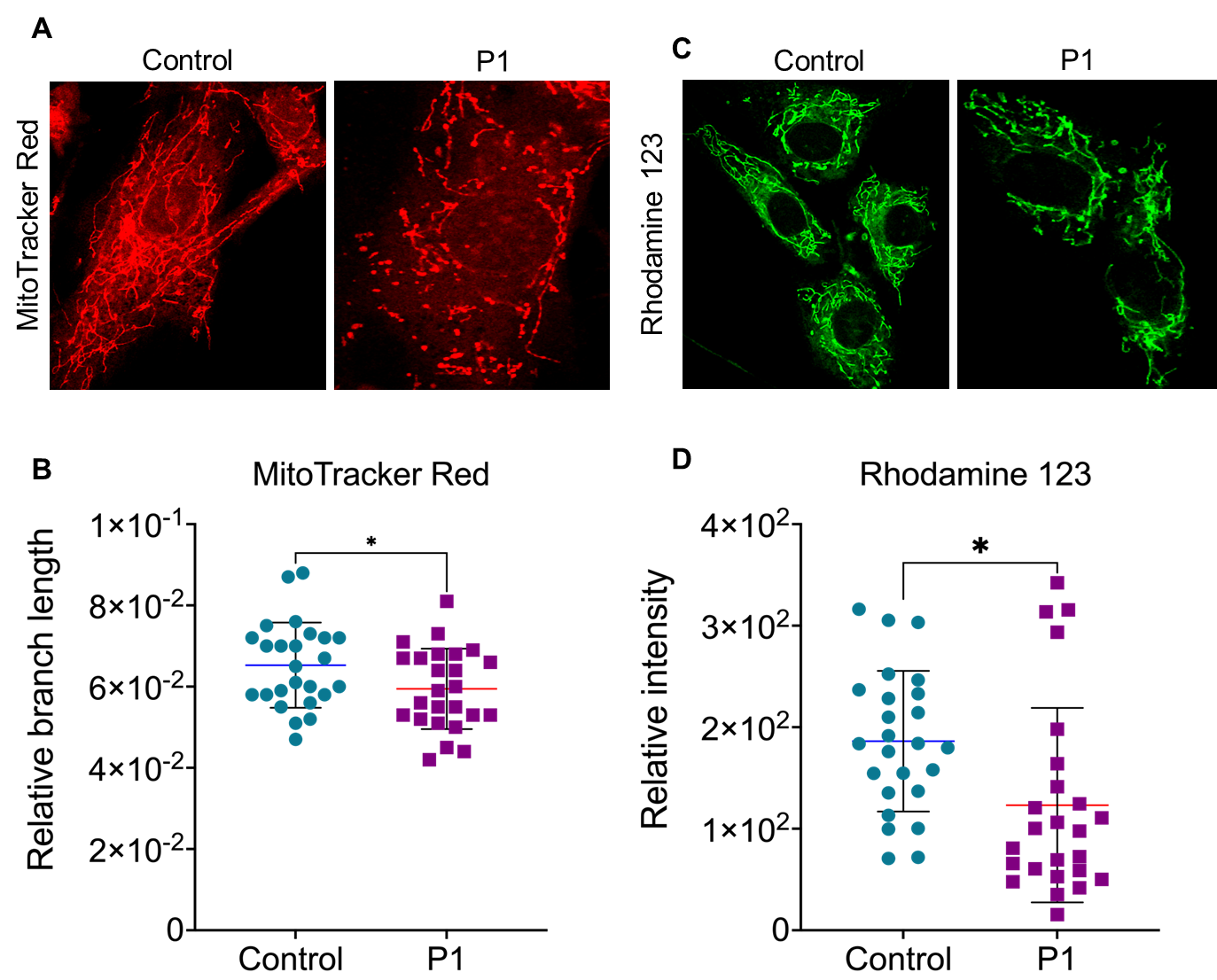


**Supplementary Figure 3 :** **(A)** Representative confocal image of control and P1 cell lines stained with MitoTracker Red showing reduced branch length in P1 **(B)** Bar graph showing the relative branch length decreasing in P1 cell lines **(C)** Representative confocal image of control and P1 cell lines stained using Rhodamine123 for mitochondrial membrane potential **(D)** Bar graph showing the reduced relative fluorescence intensity of Rhodamine123 indicating decrease in mitochondrial membrane potential, respectively in P1 cell lines due to defective OXPHOS activity. *p<0.05, **p<0.001, ***p<0.0001 and ****p<0.00001


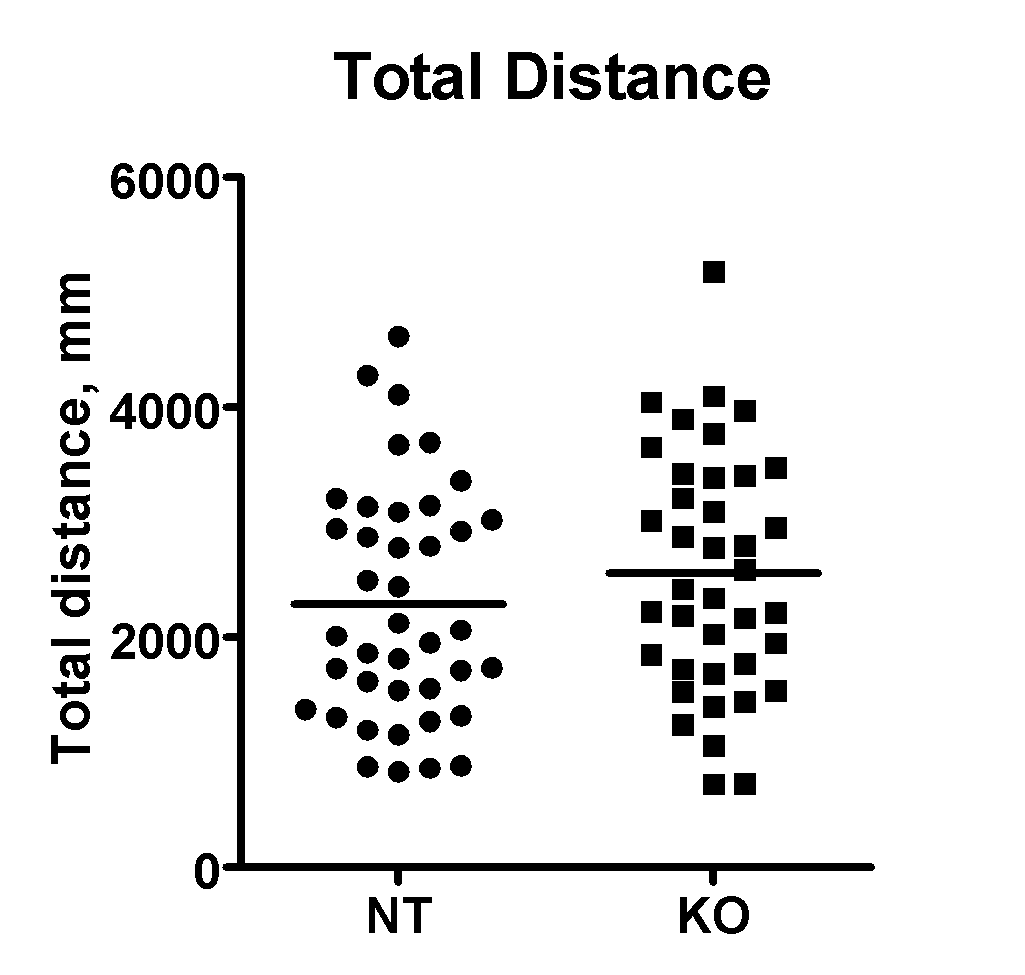

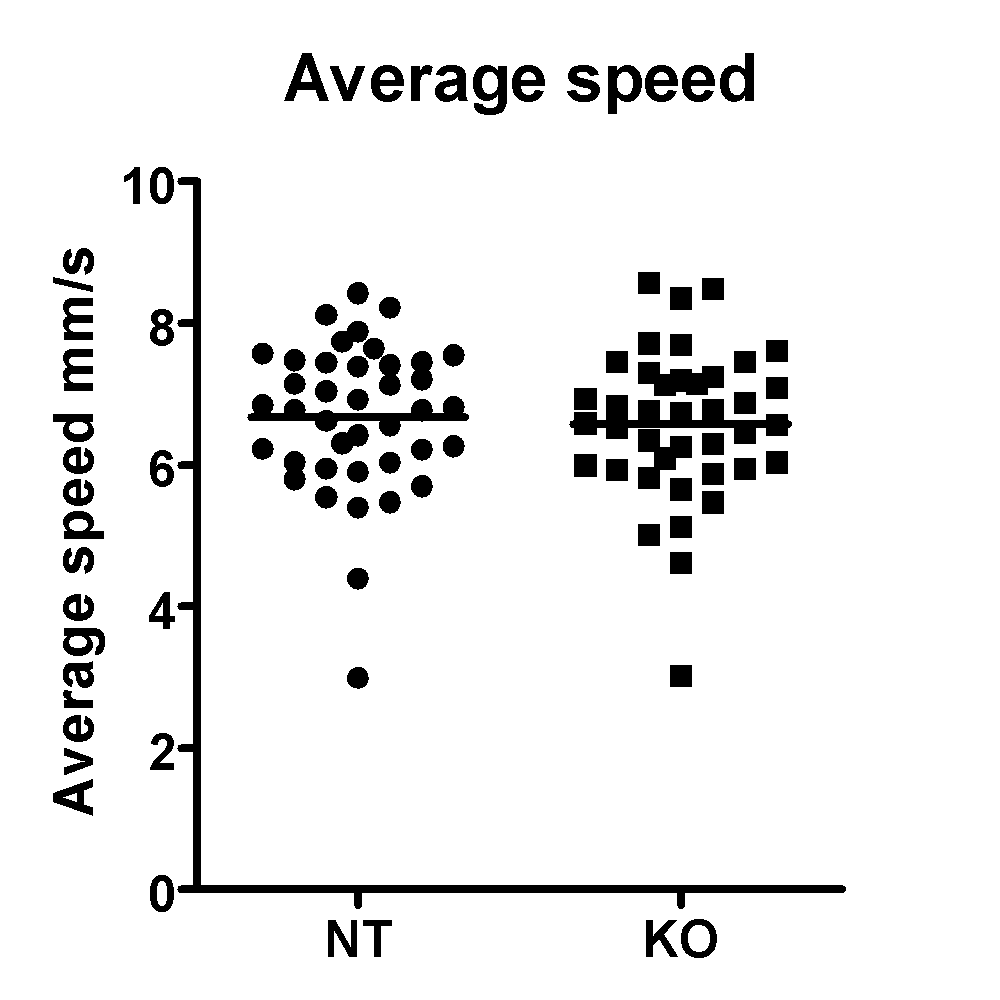


**A**

**B**


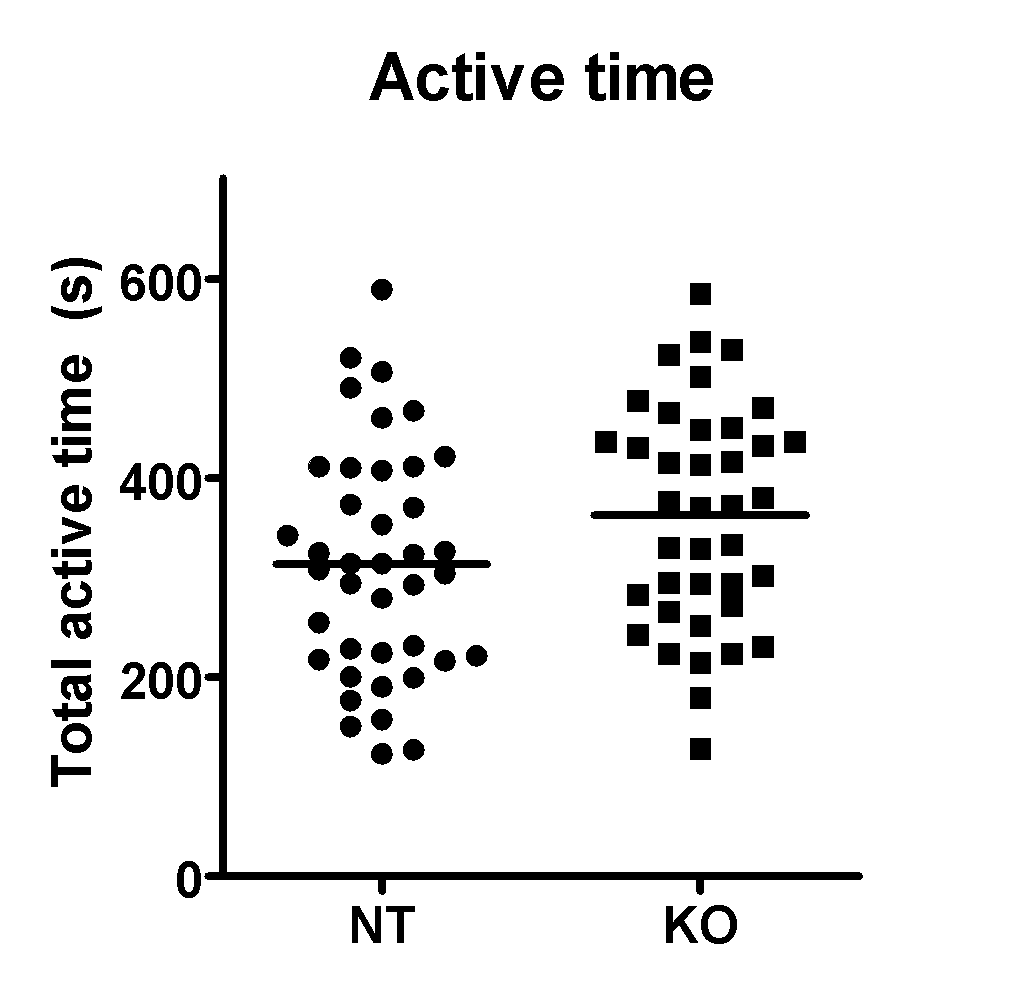

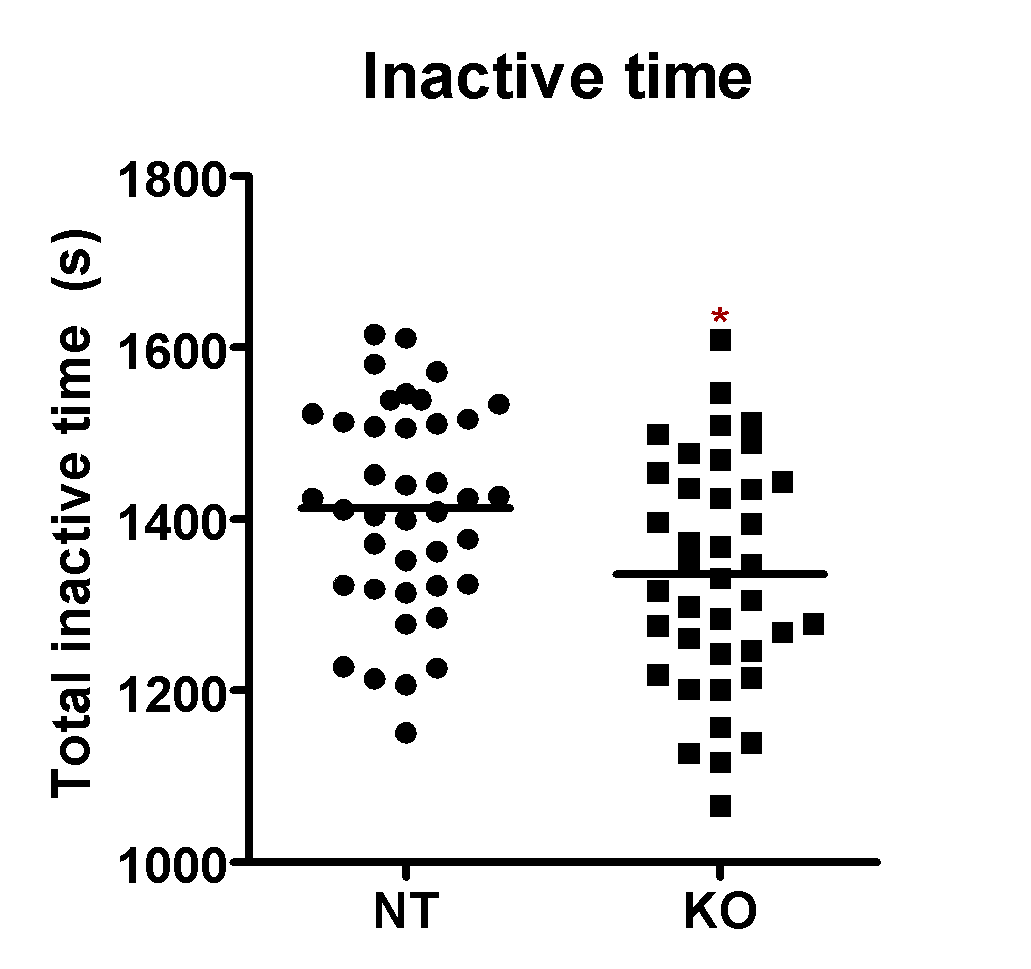


**C**

**D**

**Supplementary Figure 4: (A-D)** Analysis of various metrics of locomotion in the control and *mrsp2* crispant larvae (7dpf) including total distance moved, average speed, time spent in motion and time spent in inactivity during a standard light-dark paradigm. Data from n=40 larvae/group is shown.

**References**

1. Kuthethur R, Shukla V, Mallya S, Adiga D, Kabekkodu SP, Ramachandra L, et al. Expression analysis and function of mitochondrial genome-encoded microRNAs. J Cell Sci. 2022 Apr 15;135(8):jcs258937.

2. Valente AJ, Maddalena LA, Robb EL, Moradi F, Stuart JA. A simple ImageJ macro tool for analyzing mitochondrial network morphology in mammalian cell culture. Acta Histochem. 2017 Apr;119(3):315–26.

3. Wierson WA, Welker JM, Almeida MP, Mann CM, Webster DA, Torrie ME, et al. Efficient targeted integration directed by short homology in zebrafish and mammalian cells. eLife. 2020 May 15;9:e53968.

4. Sorlien EL, Witucki MA, Ogas J. Efficient Production and Identification of CRISPR/Cas9-generated Gene Knockouts in the Model System Danio rerio. J Vis Exp JoVE. 2018 Aug 28;(138):56969.

5. Medishetti R, Balamurugan K, Yadavalli K, Rani R, Sevilimedu A, Challa AK, et al. CRISPR-Cas9-induced gene knockout in zebrafish. STAR Protoc. 2022 Dec 16;3(4):101779.

6. Kimmel CB, Ballard WW, Kimmel SR, Ullmann B, Schilling TF. Stages of embryonic development of the zebrafish. Dev Dyn Off Publ Am Assoc Anat. 1995 Jul;203(3):253–310.

7. Sengupta A, Padhan DK, Ganguly A, Sen M. Ccn6 Is Required for Mitochondrial Integrity and Skeletal Muscle Function in Zebrafish. Front Cell Dev Biol. 2021;9:627409.

8. Rani R, Sri NS, Medishetti R, Chatti K, Sevilimedu A. Loss of FMRP affects ovarian development and behaviour through multiple pathways in a zebrafish model of fragile X syndrome. Hum Mol Genet. 2024 Aug 6;33(16):1391–405.
